# BEST PRACTICES FOR STUDYING OLDER MIGRANTS IN CONTEXT

**DOI:** 10.1093/geroni/igad104.1312

**Published:** 2023-12-21

**Authors:** Ashley Ritter, Allen Glicksman, Robert Schrauf

## Abstract

Understanding health outcomes of the older migrant (ages 60+) requires insight into the perceptions of the older person and considering the impact of the environment in which the older person lives. The four papers on this panel address these issues, each using a different methodology. Bilecen’s study, using qualitative methods, examines the impact of the wording of questions on the ways in which older Turkish migrants in the Netherlands discuss loneliness. The paper helps us understand both perceptions of older migrants and the influence of methodology used on the types of responses collected. Diederich’s paper on Cuban migrants to the United States uses census data to investigate the impact of changes in the environment of arrival at different points in time on care arrangements later in life. She demonstrates the value and limitations of using this type of data. Torres uses a survey of the scientific literature to ask questions about the impact of bigotry on the well-being of older immigrants and whether lack of attention to this topic means there is an important missing piece in our understanding of the experience of the older migrant. Glicksman et. al. use clinical data to describe interactions with the formal health and social service systems by persons of limited English proficiency, an opportunity to understand both actual service use and the benefits and limitations of using clinical data for this purpose. Schrauf’s response will discuss both the findings and the relation of methods to goals in the study of older migrants. This is an International Aging and Migration Interest Group Sponsored Symposium.

